# Ovarian Herniæ.—Their Causes, Symptoms, and Treatment

**Published:** 1890-09

**Authors:** Thomas More Madden

**Affiliations:** Physician to the Hospital for Sick Children, Dublin; Obstetric Physician and Gynæcologist Mater Misericordlæ Hospital; Examiner Conjoint Board Royal College of Surgery and Apothecaries’ Hall, Ireland; Consultant National Lying-In-Hospital; Ex-President Obstetric Sections of the Royal Academy of Medicine, Ireland, and of the British Medical Association; Formerly Vice-President British Gynæcological Society; M. D. Honoris Causa Texas Medical College, Etc.


### OVARIAN HERNIÆ.—THEIR CAUSES, SYMPTOMS, AND TREATMENT.

BY THOMAS MORE MADDEN, M. D., F. R. C. S. ED.

Physician to the Hospital for Sick Children, Dublin; Obstetric Physician and Gynæcologist Mater Misericordlæ Hospital; Examiner Conjoint Board Royal College of Surgery and Apothecaries' Hall, Ireland; Consultant National Lying-In-Hospital; Ex-President Obstetric Sections of the Royal Academy of Medicine, Ireland, and of the British Medical Association; Formerly Vice-President British Gynæcological Society; M. D. *Honoris Causa* Texas Medical College, Etc. *

Ovarian herniæ are amongst the most neglected, although clinically they should be included among the most important, troubles that come before us in gynæcological practice. In the great majority of cases they occur downwards into Douglas' space, and in such instances the left ovary is that most frequently displaced. The next in point of frequency of these herniæ are those occuring in the inguinal regions where they are either found above Poupart's ligament or is more commonly the case, follow the course of the canal of Nuck downwards, and forwards, and so present in the labia where they may be readily recognized.

In the former or directly downward variety of displacement, the ovary may be discovered on vaginal examination in the recto vaginal fossa as a small, oval-shaped, firm, elastic and highly sensitive tumour, bulging forward into the post-cervical *cul de sac.* In the larger number of cases ovarian herniæ, especially those in Douglas' space result from the *vis a tergo* of abdominal or uterine tumours, or from the tension on the appendages occasioned by displacements of the uterus.

*Diagnosis.*—Until recently these herniæ when inguinal were very generally compounded with enlarged glands ; when labial with other tumours in that situation; and when downwards with pelvic abscess, and hamatocele. Or as often happens, they are mistaken for the retroflexed fundus uteri, and the patient suffering from an ovarian prolapse is vainly treated for a nonexistent retroflexion or retroversion of the uterus. There can now be no excuse for such errors. The sudden occurrence of the tumour, its physical character, the peculiar dull sickening pain, and the extreme tenderness and nausea manifest on examination are sufficient to enable a correct diagnosis to be made by any competent gynæcologist.

*Treatment.*—Where the ovarian herniæ takes place through either of the abdominal rings or downwards in the Douglas' space, it may in some instances be reduced, as any other herniæ similar situated. In the majority of cases, however, such herniæ are irreducible when discovered, and must either be supported in the former case by applying a hollow truss whilst in the latter case the prolapsed ovary must be replaced if possible and kept in position with a peculiar form of pessary, exhibited, especially devised by Dr. More Madden for the purpose, or failing this, if the symptoms be urgent the ovary must in some cases be removed.

The foregoing views are illustruted in the paper of which this is an abstract by the details of several instances of ovarian herniæ, exemplifying the clinical history and treatment of such cases.

---

* British Medical Association, annual meeting, Birmingham, July, 1809, —Obstetric sestion—Abstract of a paper.

---

TEST FOR DRINKING WATER.—A test for the purity of drinking water is given as follows by Professor Angell, of the Michigan University:

"Dissolve about half a teaspoonful of the purest white sugar in a fruit bottle, completely full of water to be tested, tightly stopped; expose it to daylight and a temperature up to 70 degrees Fahr. After a day or two examine, holding the bottle against something black for floating specks, which will betray the presence of organic matter in considerable proportion.—*Ex.*

---

A university for Toulouse has been proposed, and the preliminaries of its establishment are said to be well under way.

